# Low-Level Contrast Statistics of Natural Images Can Modulate the Frequency of Event-Related Potentials (ERP) in Humans

**DOI:** 10.3389/fnhum.2016.00630

**Published:** 2016-12-09

**Authors:** Masoud Ghodrati, Mahrad Ghodousi, Ali Yoonessi

**Affiliations:** ^1^Department of Physiology, Monash UniversityClayton, VIC, Australia; ^2^Neuroscience Program, Biomedicine Discovery Institute, Monash UniversityClayton, VIC, Australia; ^3^Department of Neuroscience, School of Advanced Technologies in Medicine, Tehran University of Medical SciencesTehran, Iran

**Keywords:** visual perception, natural images, image statistics, visual cortex, event-related potentials

## Abstract

Humans are fast and accurate in categorizing complex natural images. It is, however, unclear what features of visual information are exploited by brain to perceive the images with such speed and accuracy. It has been shown that low-level contrast statistics of natural scenes can explain the variance of *amplitude* of event-related potentials (ERP) in response to rapidly presented images. In this study, we investigated the effect of these statistics on *frequency* content of ERPs. We recorded ERPs from human subjects, while they viewed natural images each presented for 70 ms. Our results showed that Weibull contrast statistics, as a biologically plausible model, explained the variance of ERPs the best, compared to other image statistics that we assessed. Our time-frequency analysis revealed a significant correlation between these statistics and ERPs' power within theta frequency band (~3–7 Hz). This is interesting, as theta band is believed to be involved in context updating and semantic encoding. This correlation became significant at ~110 ms after stimulus onset, and peaked at 138 ms. Our results show that not only the amplitude but also the frequency of neural responses can be modulated with low-level contrast statistics of natural images and highlights their potential role in scene perception.

## Introduction

Categorization of complex natural images is performed with stupendous speed and with outstanding accuracy by our visual systems. This happens despite multiple confounding factors including changes in illumination, contrast, and viewing distance. Early behavioral studies have revealed that humans are easily able to categorize object images within a fraction of a second with significantly high accuracy (Potter and Levy, 1969; Potter, 1976; VanRullen, 2007; Fabre-Thorpe, 2011; Mack and Palmeri, 2011). Also, event-related potential (ERP) experiments in humans have shown that the categorization of rapidly presented animal and non-animal images is achieved ~150 ms after stimulus onset (Thorpe et al., 1996; Vanrullen and Thorpe, 2001). These suggest that rapid categorization is performed through the feed-forward flow of information across ventral visual pathway and brain exploits the most relevant visual features at first glance for decision-making (Thorpe et al., 1996; Vanrullen and Thorpe, 2001; Oliva, 2005; Greene and Oliva, 2009a,b). Yet, the visual features that are extracted by brain very early after viewing an image are not well-known.

Natural images, despite being highly complex, are governed by statistical regularities and it is thought that sensory processing has been adapted to these statistical properties in natural environment (Field, 1987; Simoncelli and Olshausen, 2001; Yoonessi and Kingdom, 2008). Several studies have investigated the relationship between these natural image statistics and visual perception. For example, behavioral studies suggest that Fourier amplitude and phase spectrum of natural images provide category-related information, while images are rapidly presented (Kaping et al., 2007; Loschky et al., 2007; Honey et al., 2008; Gaspar and Rousselet, 2009; Joubert et al., 2009). Similarly, modeling studies have successfully employed spatial frequency content to categorize natural images (Torralba and Oliva, 2003; Drewes et al., 2005). It has also been shown that human reaction time and accuracy can be predicated based on natural image statistics (Groen et al., 2012b; Mirzaei et al., 2013).

Other studies provide evidence that neural responses are modulated with different statistical properties of natural images. Several studies have shown that components of early visual evoked potentials (VEP) in response to natural images are significantly modulated with different spatial frequencies of the images (Hansen et al., 2011, 2012). Also, the modulation of EEG signals in response to images with different global low-level statistics such as luminance (Martinovic et al., 2011), power spectrum (Johnson and Olshausen, 2003), and phase information (Baker et al., 2008; Rousselet et al., 2008; Bieniek et al., 2012) has been reported. Nevertheless, it is still unclear what statistical properties of natural images elicit stronger modulations on neural responses.

Recent studies show that Weibull contrast statistics can be one of the good candidates for such properties, as they follow a biologically plausible model and are highly correlated with the model responses reported for X and Y cells in Lateral Geniculate Nucleus (LGN) (Ghebreab et al., 2009; Scholte et al., 2009). Interestingly, it has been demonstrated that ERPs in response to natural images, presented to human subjects, are significantly correlated with image statistics calculated based on contrast distribution of images (Ghebreab et al., 2009; Scholte et al., 2009; Groen et al., 2012a,b, 2013). These studies showed that the contrast distribution of natural images follows Weibull distribution. Moreover, the Weibull contrast statistics are not only correlated with the amplitude of ERPs (Ghebreab et al., 2009; Scholte et al., 2009), but also are correlated with human reaction time and behavioral performance (Groen et al., 2012b, 2013; Mirzaei et al., 2013).

In this study, we extended these findings by investigating whether Weibull statistics can explain the frequency modulation of neural responses to a diverse set of natural images (Torralba and Oliva, 2003; Serre et al., 2007; Crouzet and Serre, 2011), as other studies have earlier shown that the frequency content of brain signals [e.g., local field potentials (LFP) and EEG] can be modulated by the properties of visual stimulus (Tallon-Baudry et al., 1998; Kayser and König, 2004; Henrie and Shapley, 2005; Berens et al., 2008; Freunberger et al., 2008).

To address this question, we recorded ERPs from human subjects, viewing a variety of natural images. We extracted different image statistics, including Weibull contrast statistics, from the image set and investigated their relationship with human ERPs in time-frequency domain. We showed that Weibull statistics show significant correlation between ~110–185 ms after stimulus onset within the frequency band of ~3–7 Hz (theta band), suggesting that brain may exploit such statistical regularities for rapid scene understating. The modulation in theta band is thought to be associated with context updating, episodic, and semantic encoding in different cognitive processes (Klimesch, 1999; Makeig et al., 2004; Sauseng et al., 2007). We further supported these results using information theoretic analysis as a more robust method beyond correlation analysis.

## Materials and methods

### Stimulus set

The stimulus dataset included 1000 images, divided into two groups of animal and non-animal images, each with 500 images. The animal category contained images of a wide variety of animals in their natural habitat. Half of the non-animal images (250) were natural environments (e.g., moor, mountain, forest, and river), and the other half were images from man-made environments (e.g., buildings and streets). There were four sub-categories in each major category including: Head, close-body, medium-body and far-body (representing the viewing distance from the camera). Each sub-category contains 125 images. The size of the images was 256 × 256 pixels. The image database is publicly available [http://cbcl.mit.edu/software-datasets/serre/SerreOlivaPoggioPNAS07/index.htm, collected, by Torralba and Oliva (2003)].

### Participants and image presentation

EEG signals were recorded from 13 human subjects (aged 26–36; mean age 30, all male) while viewing images. Subjects had either a normal or corrected-to-normal vision. Participants sat on a chair located in a room under dim light condition at a viewing distance of 57 cm from the computer screen. The stimuli were presented on a 17″ CRT monitor (mean luminance 61 cd/m^2^) with 1024 × 768 pixels resolution and 85 Hz refresh rate using MATLAB and psychophysics toolbox (Brainard, 1997; Pelli, 1997) (http://psychtoolbox.org).

**Figure 1:**
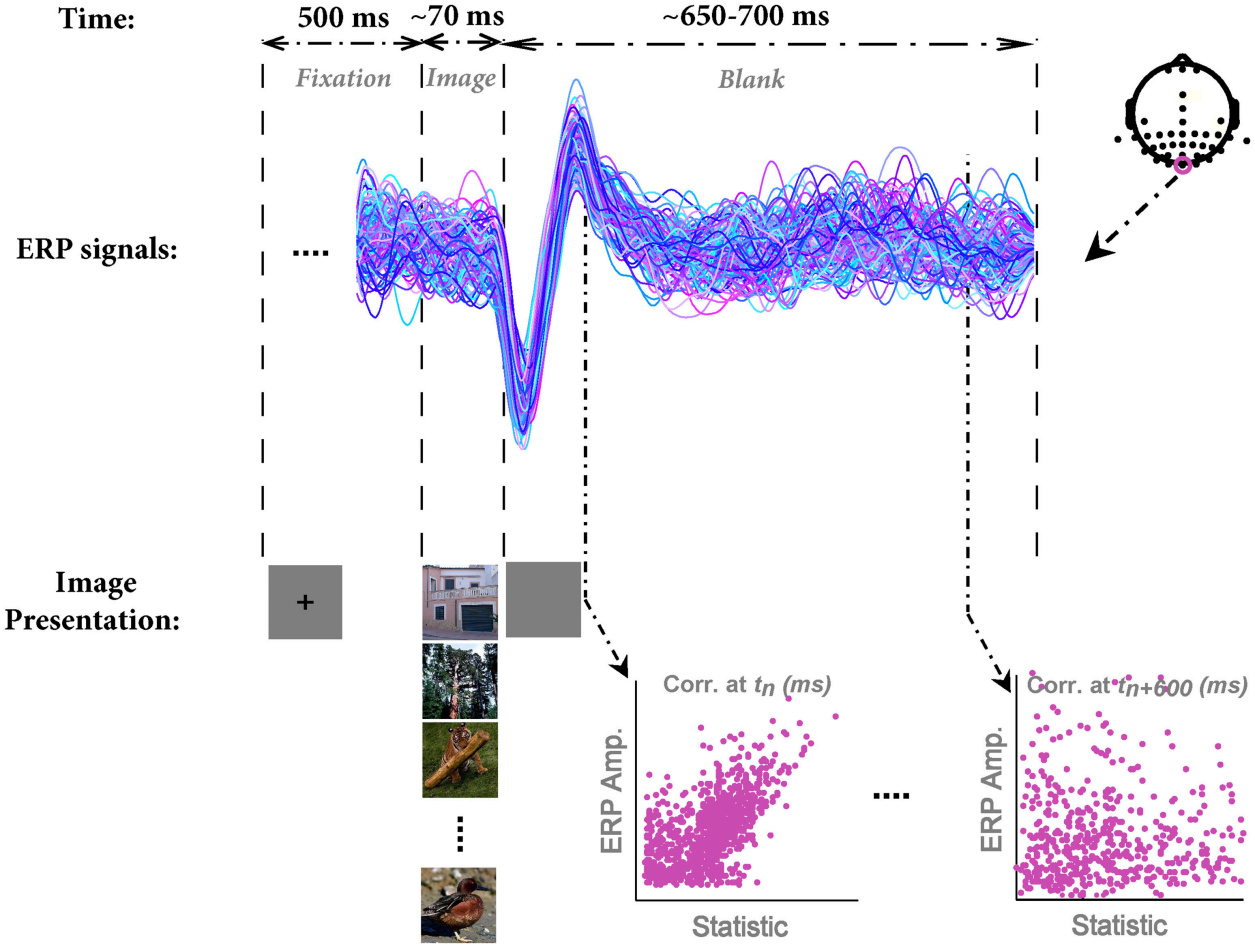
**The paradigm of stimulus presentation, sample ERPs, and the method for correlation analysis**. In each trial, a fixation cross was presented for 500 ms; then an image was randomly selected from the image database and presented to subjects for the duration of 70 ms (6 frames, monitor refresh rate 85 Hz). Note that we used grayscale images in the actual experiment; here, we showed them in color for the sake of better visualization. Subsequently, a blank screen was presented for ~650–700 ms. Next trial was started immediately after the end of blank screen. EEG signals were segmented into epochs of 800 ms (from 100 ms before stimulus onset to 700 ms post-stimulus). ERPs are shown for a sample subject for *O*_*z*_ electrode. Each color shows a single trial ERP in response to an image. The electrode positions are shown at the top-right corner. We recorded from 42 electrodes, mostly located on occipital and temporal lobe. The correlation between each image statistic and ERPs was calculated across all time points, subjects, and electrodes.

In each trial, a fixation cross was presented at the center of the screen for 500 ms. Subsequently, an image was randomly selected from the database and presented for the duration of ~70 ms (6 frames in 85 Hz monitor) centrally positioned on the screen (covering ~11°^*^11° of visual angle) against a gray uniform background. A blank screen was then presented for ~650–700 ms as inter-stimulus interval (ISI). Participants were instructed to maintain their fixation throughout the trial and see whether the image contained any animal or not (Figure 1). The next trial was started immediately after the blank period. Images were drawn without replacement, meaning that they presented once to each subject.

All images were converted to grayscale. This was done to remove any possible color effect. This also allowed us to calculate image statistics on a single color channel (i.e., gray). Images were divided into 4 blocks, each containing 250 images from the dataset with the same number of animals and non-animals in each block. All images in each block were randomly selected and presented. Subjects required approximately 6 min for completing each block. After finishing each block, we encouraged them to take a rest. Each experiment (4 blocks) took around 40 min in total.

All subjects voluntarily participated to the experiments and gave written informed consent prior to participation. All experimental protocols were approved by the ethical committee of the Institute for Cognitive Science Studies (ICSS). Experiments were carried out in accordance with the guidelines of the declaration of Helsinki and the ethical committee of the Institute for Cognitive Science Studies (ICSS).

### Image statistics

#### Extracting weibull contrast statistics from images

**Figure 2:**
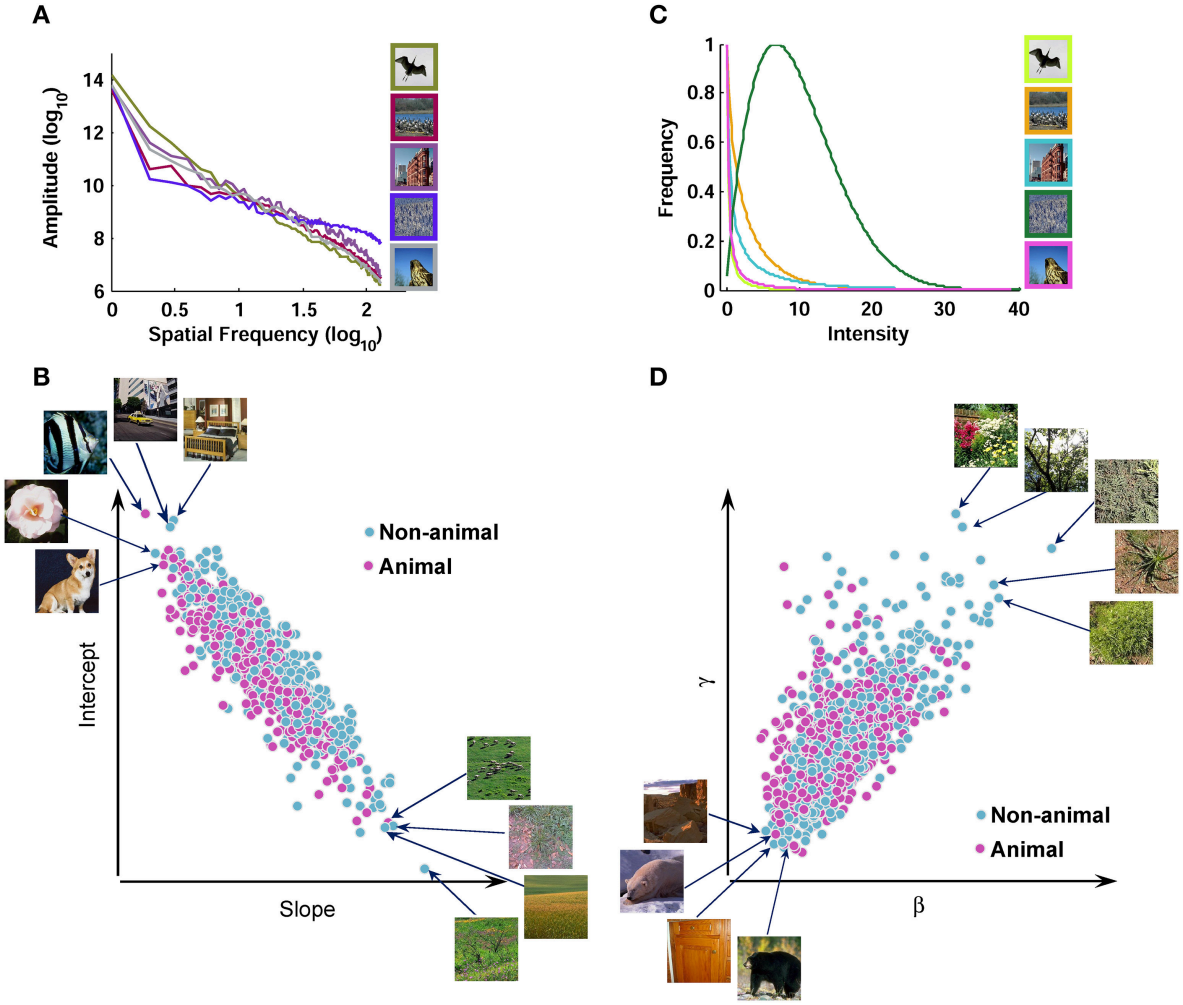
**Scatter plot of different image statistics for the database. (A)** Fourier amplitude spectrum of several sample images from the image database. Note that all image statistics were calculated using grayscale images; here, we showed them in color for the sake of better visualization. The amplitude spectrum has a negative slope for natural images. A line was fit to the amplitude spectrum and the slope and intercept were used as Fourier amplitude statistics. **(B)** Scatter plot of slope against intercept for all images. **(C)** Contrast distributions for several sample images after applying LGN-like filters and filter selection (Materials and Methods). The contrast distributions follow Weibull distribution. A Weibull function was fit to the contrast distribution and two parameters (i.e., beta and gamma) were extracted as Weibull contrast statistics. **(D)** Scatter plot of beta against gamma for all images.

It has been suggested that the contrast distribution of natural images follows Weibull distribution. Weibull distribution has two free parameters that change the scale (beta) and the shape (gamma) of the distribution (Simoncelli, 1999; Ghebreab et al., 2009). The beta parameter varies with the range of contrast strengths in the image, whereas gamma varies with the degree of correlation between contrasts (scene fragmentation). We extracted Weibull contrast statistics using a method similar to Groen et al. (2012b). First, gray-scale images were filtered with a set of biologically plausible LGN-like spatial filters (Bonin et al., 2005). They were second-order derivative of Gaussian filters with different sizes (Bonin et al., 2005; Ghebreab et al., 2009). It has been shown that for the estimation of gamma parameter larger filter sizes are more appropriate, while the beta parameter is well-estimated using smaller filter sizes (Scholte et al., 2009). Therefore, for the beta parameter, a bank of filters with 5 octave scales (4, 8, 16, 32, and 64 standard deviation in pixels) was used; for the gamma parameter, the filter bank consisted of octave scales 5, 10, 20, 40, and 80. Following Groen et al. (2012b), we normalized the output of each filter using a Naka-Rushton function, consisting of 5 semi-saturation constants (from 0.15 to 1.6) that met the spectrum from linear to non-linear contrast gain control in the LGN. After applying the filter banks, we selected the smallest filter with the output higher than what is expected to be noise for that specific filter for beta and gamma, using minimum reliable scale selection (Elder and Zucker, 1998). The noise thresholds were computed using a large pool of natural images (Groen et al., 2012b). After selecting an appropriate filter response, the result was a contrast magnitude map with the size of 256 × 256 pixels. Finally, a Weibull function was fitted using maximum likelihood estimation (MLE-with 96% confidence) to the histogram of the contrast maps, to determine beta and gamma parameters, which were used as Weibull contrast statistics (Figure 2).

#### Extracting fourier statistics from images

We also calculated Fourier statistics based on the frequency content of the images, using an approach similar to Oliva and Torralba (2001). Each gray-scale image was transformed to frequency domain using 2-dimentional Fast Fourier Transform. Then, a 1-dimentional curve was obtained by rotational averaging on the amplitude spectrum of each image. Finally, a line was fit to the log-log representation of this curve, providing us with the two Fourier statistics: Slope and Intercept (Figure 2).

#### Global luminance-contrast statistics

To make our results comparable to previously reported results (Scholte et al., 2009), we calculated Michelson and Root Mean Square (RMS) contrasts as two other standard contrast measures. Michelson contrast was obtained using: *M* = *L*_*max*_ − *L*_*min*_*/L*_*max*_ + *L*_*min*_, where *L*_*min*_ is the lowest intensity and *L*_*max*_ is the highest intensity in the image. The RMS contrast was calculated by dividing the standard deviation of the intensity values of all pixels in the image to the mean intensity. The average Michelson contrast of the images was 99 ± 4% (mean ± STD; minimum = 50%, maximum = 100%), while the average RMS contrast of the images was 0.52 ± 0.17 (mean ± STD; minimum = 0.12, maximum = 1.62).

### Data acquisition and pre-processing

EEG signals were recorded using a 64-channel data acquisition system (Asalab, 64-channel EEGẼRP recording device manufactured by ANTneuro, Netherland). We used an extended 10–20 standard cap for recording. Signals were recorded at 512 Hz sampling rate. We recorded from 42 channels, mostly located in occipital and temporal areas (see Figure 1, right inset), since early recordings and analyses indicated that channels in frontal part were not strongly modulated with our visual stimulation. Moreover, previous studies showed that only occipital channels were strongly correlated with image statistics (Scholte et al., 2009; Groen et al., 2012a,b, 2013). Offline referencing was performed using two earlobe electrodes. Eye movements rarely affected the signals recorded on occipital electrodes; however, we recorded from 3 frontal electrodes, and removed the effects of eye movements on EEGs using thresholding. Offline filtering was performed using MATLAB filter design. For the amplitude analyses, signals were first filtered with a high-pass filter with cut-off frequency of 0.1 Hz and then with a low-pass filter with cut-off frequency of 30 Hz. For the frequency analyses, signals were filtered between 0.1–60 Hz (details are provided in the next section). All signals were segmented from 100 ms before stimulus onset to 700 ms after stimulus presentation. We used −100 to 0 ms of each epoch for baseline correction, while 0 ms representing stimulus onset. To have more reliable ERPs, we used an automatic artifact removal method for removing outliers. For this purpose, we first calculated a global average for all ERPs recorded from each channel, separately for each subject. Then, we measured the distance (Euclidean distance) between each recorded ERP (related to each stimulus) and the global averaged ERP (separately for each subject and electrode). Each ERP that was deviated more than ±3 STD from the mean was removed from further analysis. The minimum rejection rate was 10% with the maximum of 21% (median 13%). Other measures, such as correlation, were also tested but Euclidean distance showed the most reliable result. Finally, all signals were converted to Current Source Density (CSD) responses to have more localized signals (Perrin et al., 1989). Note that although CSD made the recorded signals more localized around each electrode (Nunez and Srinivasan, 2006), it did not change the results (i.e., the strength of correlation; described later). This way allowed us to find which electrode has the strongest modulation driven by stimulus. All analyses were performed in MATLAB using custom-made programs (MathWork, on a 3 GHz CPU, 64 Bits desktop computer).

### Data analysis

#### Correlation with ERP amplitude

To assess whether different image statistics can explain the modulations in the amplitude of ERPs, we calculated the Pearson's correlation between each image statistics and ERPs for every electrode, time point, and subject, separately (Figure 1). First, all single trial ERPs in response to images were loaded into MATLAB with their corresponding image statistics. Then, we computed the correlation between image statistics (e.g., 1000 beta parameters) and ERPs' amplitude at each time point (from −100 to 700 ms). This provided us with a correlation coefficient and a *p*-value at each time point. To correct for multiple comparisons (time points, electrodes and parameters), *p*-values were FDR-corrected at = 0.05. To test whether the combination of image statistics can explain higher variance compared to using each image statistic individually, we ran two other regression analyses: (1) Using all image statistics as regressors on ERP amplitude. We called this *Full model*. (2) Using both Weibull parameters as regressors. This model was called *Weibull model*.

#### Correlation with ERP frequency

The main focus of our study was to see whether the differences in images statistics can explain the modulations in the power of different frequencies of ERPs. Note that all frequency analyses were performed on ERPs filtered between 0.1 and 60 Hz. Also, we subtracted the average ERP from the single trials for our frequency analysis. This way we removed the phase-locked ERP component from the overall EEG signal. Then, each single trial ERP was transformed to Fourier space and the power spectrum of the ERP was computed (for every channel and subject separately). Having done this, we generated a power spectrum for each single trial ERP. Subsequently, the correlation between each image statistic and the power of each frequency was calculated, providing us a correlation coefficient and a *p*-value for that frequency. Note that this stage is similar to the correlation analysis for ERP amplitude, but here we have power spectrum instead. To correct for multiple comparisons (frequencies, electrodes, and parameters), *p*-values were FDR-corrected at = 0.05.

In order to investigate the time course of the modulation of power at each frequency with image statistics, we calculated the spectrogram of ERPs. First, we generated a spectrogram for each single trial ERP (e.g., see **Figure 7A** for the average spectrogram). After calculating the spectrograms of single trial ERPs, we obtained the correlation between the image statistics and the power in the time-frequency domain. This way, we generated a correlation matrix for each subject in which each row indicates the correlations with a specific frequency at different time points (columns are time points: 200 ms before stimulus onset to 800 ms after). The reported results are the average correlation matrix of all subjects (**Figure 7B**). Spectrograms were calculated using a sliding window with the size of 150 ms and steps of 20 ms.

#### Mutual information

We also calculated the mutual information (MI) between the ERPs and the image statistics using Equation 1.

(1) $$\begin{array}{r} MI\left(S,R\right)=\underset{s\in S}{\sum }p\left(s\right)\underset{r\in R}{\sum }p\left(r|s\right)\log_{2}\left(\frac{p\left(r|s\right)}{p\left(r\right)}\right) \end{array}$$

Where *R* is the amplitude of ERPs in a sliding time window, *S* is the image statistics (e.g., beta parameter), *p(s)* is the probability of presenting stimulus *s* with corresponding statistics, *p(r)* is the probability of observing response *r* evoked by stimulus, *p(r|s)* is the conditional probability of observing response *r* given stimulus *s* was presented. To calculate MI, we considered a sliding time window on ERPs with the size of 20 ms and step of 10 ms. We then binned the ERPs amplitude as well as image statistics into 100 bins. The bin number and the size of sliding window were found by testing different values for these parameters. The MI was then calculated from 100 ms before stimulus onset to 700 ms after. To find the time points where MI was significant, we used a shuffling test and found the time points when MI significantly deviated from the shuffled MI (Wilcoxon rank-sum test). The shuffling test was performed by calculating MI between image statistics and shuffled ERPs. This was repeated for 50 times and 95% confidence interval of the resulting distribution was calculated at each time point.

## Results

**Figure 3:**
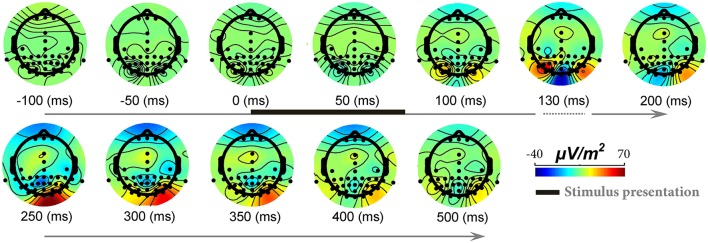
**The modulation of EEG signals in response to natural images across different electrodes and time points**. Each topographic map shows ERPs modulation across different electrodes, from 100 ms before stimulus onset to 500 ms post stimulation (for a sample subject). The color-coded topographic plots demonstrate the amplitude of signals specified with a color bar at the right end. The timing for stimulus presentation is specified with a thick black line at top row (from 0 to ~70 ms). Modulations start from >110 ms after stimulus presentation and end at ~250 ms. Note that the timing is not evenly spaced between 100 and 200 ms since we aimed to show the maximum modulation time that is ~125–130 ms.

To show signal modulation across all recorded electrodes, we generated the topographical representations for the ERPs. Figure 3 shows this representation for one subject and illustrates how signals were modulated across 42 electrodes (also see Video S1). It can be seen that the strongest modulations were present mostly on occipital electrodes with the highest for *O*_*z*_ electrode (located at occipital midline). Earlier, we showed the ERPs for this subject (*O*_*z*_ electrode) in Figure 1. It can be seen that for this subject, the modulation of the responses began >110 ms after stimulus onset (with a negative peak) and continued until ~250 ms after stimulus onset (positive peak). Similarly, for all other subjects *O*_*z*_ electrode showed the strongest modulation started at 130 ± 25 ms after stimulus onset.

### Weibull statistics explain the variance of ERPs amplitude in response to natural images

**Figure 4:**
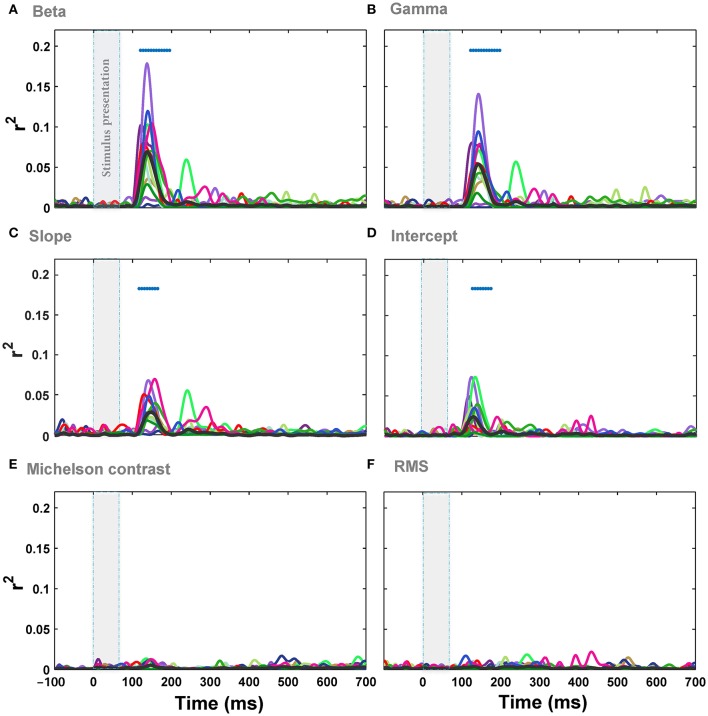
**Correlation (*r*^2^) between ERPs recorded from *O*_*z*_ electrode and different image statistics across different time points. (A)** The correlation between beta parameter and the amplitude of ERPs, recorded from *O*_*z*_ electrode, across different time points. Colors show different subjects and the average correlation across subjects is depicted using a thick black line. The significant correlation is illustrated using blue horizontal dots at the top of each plot for different times (FDR-corrected). The vertical, transparent band in each plot shows the timing for stimulus presentation. **(B–F)** Correlation between the amplitude of ERPs and gamma, slope, intercept, Michelson contrast, and RMS contrast, respectively.

We correlated individual image statistics with the amplitude of ERPs across time for every subject (see Materials and Methods). The results showed that there is a significant correlation between the image statistics and ERPs amplitude in a time window 125 ± 5 ms after stimulus onset. The maximum correlation between beta parameter of Weibull statistics and the ERPs across subjects was *r*^2^ = 0.18 (Figure 4A; minimum *r*^2^ = 0.012; mean *r*^2^ = 0.075 across all subjects at 138 ms post-stimulus onset, *p-value* < 0.0001, FDR-corrected). For gamma parameter of Weibull statistics, the maximum correlation was *r*^2^ = 0.15 (Figure 4B; minimum *r*^2^ = 0.011, mean *r*^2^ = 0.05 across all subjects at 140 ms post-stimulus onset, *p-value* < 0.0001). The maximum correlation between ERPs and Weibull statistics was observed at 138 ms after stimulus onset for beta (Figure 4A) and 140 ms after stimulus onset for gamma (Figure 4B) parameters. The correlations were measured at electrode *O*_*z*_ similar to previous studies (Scholte et al., 2009; Groen et al., 2012a,b, 2013). The average correlation between beta statistics and the ERPs amplitude across subjects and electrodes also shows that electrode *O*_*z*_ had the highest correlation (Figure S1 and Video S2).

We also assessed the correlation between Fourier statistics, i.e., slope and intercept, and the amplitude of the ERPs. Figure 4 shows the correlation between ERPs and slope (Figure 4C) as well as intercept (Figure 4D) across time for *O*_*z*_ electrode. Similar to the results of Weibull statistics, the electrode *O*_*z*_ showed the highest correlation with the maximum of *r*^2^ = 0.072 for slope (minimum *r*^2^ = 0.008, mean *r*^2^ = 0.03 at 138 ms after stimulus onset, *p-value* < 0.001) and *r*^2^ = 0.075 for intercept (minimum *r*^2^ = 0.006; mean *r*^2^ = 0.025 at 138 ms after stimulus onset, *p-value* < 0.05). These results replicate the findings of previous studies that reported a stronger mean correlation with slope compared to intercept (Groen et al., 2012a,b, 2013), although the correlations reported here are relatively weaker. We also calculated the correlations between ERPs and two other standard contrast measurements: Michelson contrast (Figure 4E) and Root Mean Square (RMS) contrast (Figure 4F). As expected, these two image statistics were not significantly correlated with the ERPs. This shows that global luminance-contrast cannot explain the variance of modulations of ERPs in our experiment. Taken together, the Weibull statistics explained the modulation of the responses better than the other single statistics examined, with beta parameter being a better predictor compared to gamma.

To address whether the combination of image statistics can explain higher variance of ERPs amplitude compared to individual statistics, we performed two additional linear regression analyses. First, we used all the statistics as regressors on ERPs' amplitude (*Full model*). Then, we used both Weibull statistics (*Weibull model*) together as regressors. The results showed that the performance of *Full model* was very similar to *Weibull model* (see Figure S2). Also, Weibull contrast statistics individually explained the variance in the responses the best, compared to any other model, supporting single parameter modeling and the corresponding explained variance.

**Figure 5:**
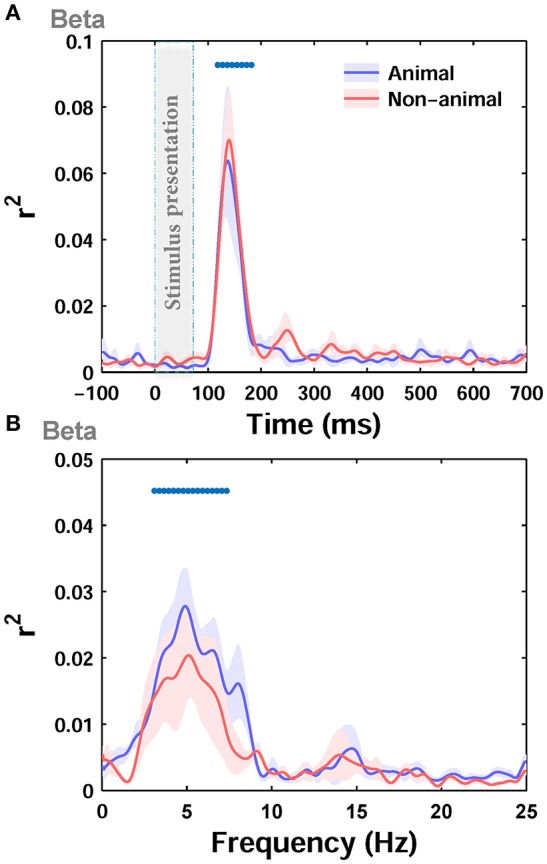
**Correlation (*r*^2^) between ERPs of animal and non-animal images and beta statistic across different time points and Frequencies**. **(A)** The average *r*^2^ over subjects between ERPs amplitude and beta parameter for animal (blue curve) and non-animal (red curve) images (recorded from *O*_*z*_ electrode). The shaded area is standard error of the mean (S.E.M). **(B)** The average *r*^2^ between beta parameter and power in different frequencies in ERPs for animal and non-animal images.

Next, we separately calculated the correlation between the ERPs and the image statistics of the two categories of images in our dataset, i.e., animal and non-animal images, as these two categories have different statistical properties. The correlation was calculated between beta parameter of Weibull statistics, as the best predictor, and the ERPs. Interestingly, the maximum correlation for the animal category (*r*^2^ = 0.17) was lower than the non-animal category (*r*^2^ = 0.275). However, the correlations were not significantly different from each other for the two categories (Figure 5A; *p* > 0.05 Wilcoxon rank-sum test). The difference in maximum correlation can be due to different statistical properties between animal and non-animal images (e.g., Johnson and Olshausen, 2003; Gaspar and Rousselet, 2009). As can be seen from visual inspection, the correlation in the animal category (Figure 5A) had lower between-subject variability compared to non-animal category (Figure 5A), suggesting that animal images shared similar features that induced similar effects on the ERPs.

### Is frequency of ERPs modulated by image statistics?

**Figure 6:**
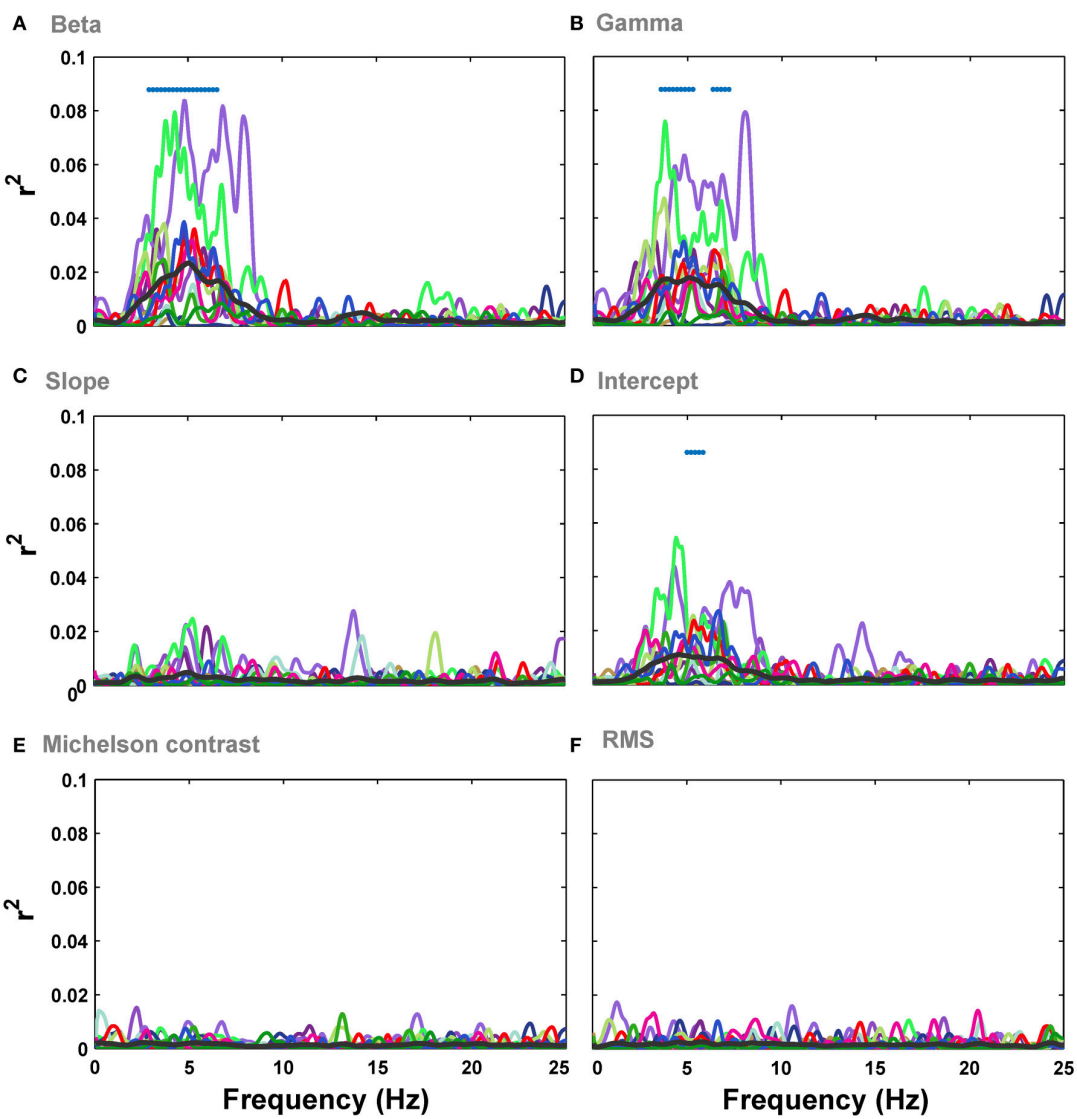
**Correlation between image statistics and the power in different frequencies of ERPs. (A)** Correlation between beta parameter and power in different frequencies in ERPs, recorded from *O*_*z*_ electrode. Colors show different subjects. The average correlation across subjects is depicted using a thick black line. Significant correlation is illustrated using a blue horizontal line at the top of each plot for different time points (FDR-corrected). **(B–F)**. Correlation between the power in different frequencies of ERPs and gamma, slope, intercept, Michelson contrast, and RMS contrast, respectively.

We then investigated the relationship between the modulation of ERPs' frequency and the image statistics. We calculated the correlation of image statistics with the power of different frequencies of ERPs. The results represented that there is a significant correlation between some image statistics and power at some frequencies in ERP signals (Figure 6). Similar to the results of correlation for ERPs amplitude, the beta and gamma of Weibull statistics showed the highest correlation with the power of ERPs, compared to the other statistics, being significant for frequencies between ~3 and 7 Hz which correspond to theta band (see Discussion). The maximum correlation observed for beta parameter of Weibull statistics was *r*^2^ = 0.08 at ERP frequency of 5 Hz (mean *r*^2^ = 0.022) and for gamma parameter of Weibull statistics was *r*^2^ = 0.078 at frequency of 7 Hz (mean *r*^2^ = 0.019). We observed a significant correlation for Intercept for frequencies between 5 and 7 Hz (Figure 6D). No significant correlation was observed for slope, Michelson, and RMS contrasts (Figures 6C,E,F).

Regression analysis for *Full model* and *Weibull model* again confirmed that the variance was explained the best by Weibull statistics compared to all other models (Figure S2). The topographic representations of correlation across electrodes and frequencies for different image statistics showed that electrode *O*_*z*_ had the highest correlation (see Figure S3 for beta parameter of Weibull statistics).

Next, by dividing ERPs into responses to animal and non-animal images, we calculated the correlation between beta parameter of Weibull statistics and the power of ERPs at different frequencies for each category of images separately. The maximum correlation was lower for the animal category (*r*^2^ = 0.08) compared to the non-animal category (*r*^2^ = 0.1), consistent with the results shown in Figure 5A for amplitude. However, the correlations, calculated for the two categories, were not significantly different from each other (Figure 5B; *p* > 0.05, Wilcoxon rank-sum text). Here again, the correlation was significant for frequencies between ~3 and 7 Hz for individual categories.

**Figure 7:**
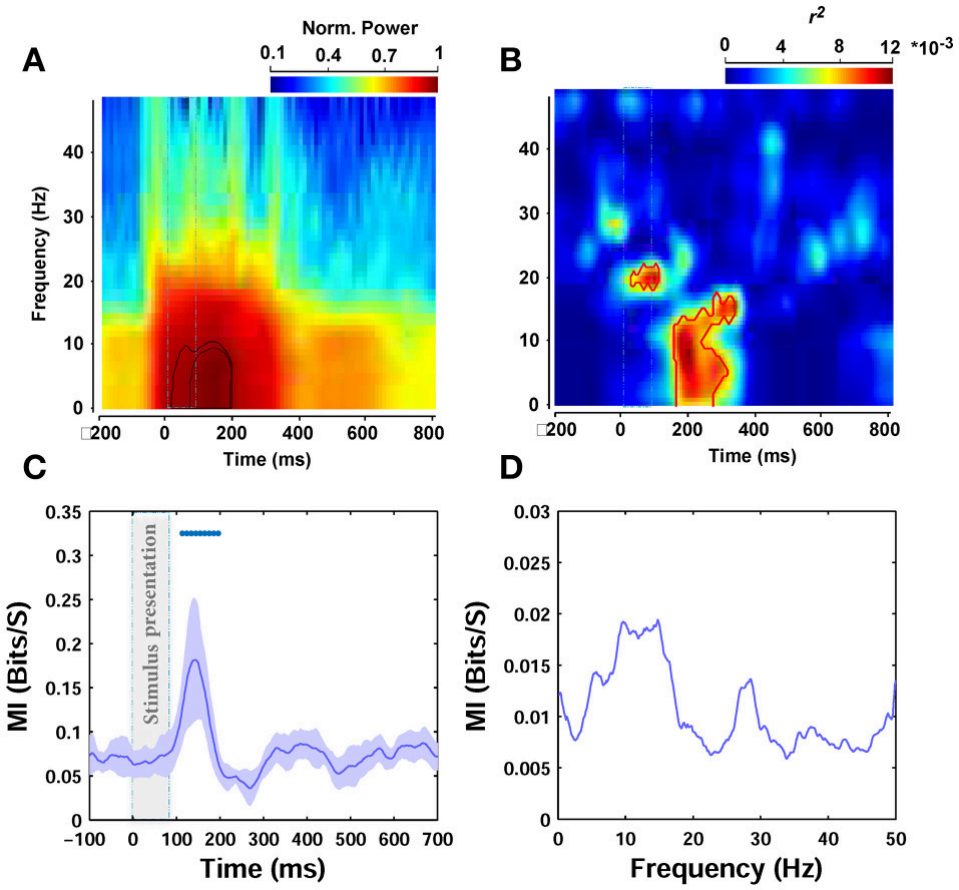
**Time-frequency and mutual information analyses. (A)** Normalized power spectrogram from 200 ms before to 800 ms after stimulus presentation. The dashed rectangular shows stimulus presentation and the contours indicates the area of maximum power. **(B)** Correlation in time-frequency domain for beta parameter. The area that is surrounded by the red contour indicates significant correlation. **(C)** MI between beta parameter and ERPs amplitude in different time points (*O*_*z*_ electrode). The average MI across subjects is depicted using a thick pink line. The shaded gray area is standard deviation (STD). **(D)** MI between beta statistic and power in different frequencies of ERPs, averaged across subjects.

To further assess the modulations at different times and frequencies, we performed a time-frequency analysis (see Materials and Methods). Spectrograms of ERPs, calculated 200 ms before to 800 ms after stimulus onset, showed an increase in power after stimulus onset between ~0 and 9 Hz (Figure 7A), consistent with the results of the frequency correlation analyses reported earlier. The power in this band peaked between ~60 and 200 ms post stimulation (see the contours plot in Figure 7A—note that the spectrograms and all frequency analyses were calculated on filtered signals between 0.1 and 60 Hz). Figure 7B represents the correlation in time-frequency domain for the beta parameter of Weibull statistics. For theta frequency band of ERPs, which showed the highest correlation, this correlation increased ~170 ms after stimulus onset and peaked at 200 ms (note the peak correlation between 0 and 12 Hz). There is also a significant increase in correlation just after stimulus onset between ERP frequencies 18–22 Hz that lasts throughout stimulus presentation. The area inside the red contour in Figure 7B shows the significant correlations.

### Significant mutual information between weibull statistics and ERPs

We also calculated the mutual information between image statistics and the ERPs, as a more robust measure, to assess the relationship between image statistics and brain signals compared to Pearson correlation. Here, we only report the mutual information (MI) between beta parameter of Weibull statistics and ERPs, as previously, beta parameter showed the highest correlation with amplitude and frequency. Figure 7C shows the results for amplitude analyses. As can be seen, the average MI across subjects significantly increased 110 ms after stimulus onset and maximized at 138 ms, consistent with the results of our correlation analyses reported earlier. Figure 7D illustrates the results for frequency analyses. The MI calculated for the power of different frequencies of ERPs was again the highest for theta frequency band. However, this increase did not reach significance for any of the frequencies examined.

## Discussion

Categorization of complex natural images is performed quickly and accurately by humans and non-human primates (Thorpe et al., 1996; Fabre-Thorpe et al., 1998; Vanrullen and Thorpe, 2001; Rousselet et al., 2002; Kirchner and Thorpe, 2006). The visual features that facilitate the rapid categorization are hotly-debated. Recently, Weibull contrast statistics have shown to be correlated with behavioral performance and the amplitude of ERPs in response to natural images in humans (Ghebreab et al., 2009; Scholte et al., 2009; Groen et al., 2012a,b, 2013; Mirzaei et al., 2013). It has also been shown that Weibull statistics can provide useful diagnostic information about global scene properties, such as naturalness (Groen et al., 2013). In this study we investigated the modulation of the frequency content of the ERPs in response to these statistics in order to extend these studies. We found a significant correlation between the modulation of theta frequency band of ERPs and Weibull statistics in the human subjects who were presented with natural images. Theta frequency band is believed to be involved in encoding new information, episodic, and semantic encoding (Klimesch, 1999; Yordanova et al., 2002; Makeig et al., 2004; Sauseng et al., 2007), although this type of modulation was mostly reported for cognitive processes (Basar et al., 2001) and not visual perception. Also, we found a significant but relatively weaker correlation in the frequency band of 18–22 Hz during stimulus presentation that falls within beta band on ERPs, which is thought to be related to visual attention (Mundy-Castle, 1951; Wróbel, 2000; Kaminski et al., 2012; Gola et al., 2013).

In this study, we also found a significant correlation between the amplitude of ERPs and Weibull contrast statistics, consistent with the findings of another group (Scholte et al., 2009; Groen et al., 2012a,b, 2013). The correlation in our study was not as strong as what reported by this group. This is potentially due to the differences in image dataset, experimental paradigm, and subject variability.

Regarding differences in databases, our image database was selected from a well-studied natural image dataset (Torralba and Oliva, 2003; Serre et al., 2007; Crouzet and Serre, 2011) that contained different animal images, man-made, and natural scenes. The first paper of this group (Scholte et al., 2009) used a subset of images derived from the Corel database, which has been reported to be biased (Wichmann et al., 2010). Subsequently, they generated a set of naturalistic images using highly controlled manipulations that resulted in several categories in terms of statistical properties (Groen et al., 2012b), which consequently resulted in a high correlation value. The correlation they reported later when they used natural images (from natural and man-made environments) (Groen et al., 2013) was lower compared to their previous studies. The database used in this last study (Groen et al., 2013) was the most similar to ours. They used a new stimulus set obtained by combining a number of different databases, but their database was not as balanced as the one used in our study (e.g., lacked close views of animals, etc.).

Another contributing factor to different correlations reported could be the difference between the tasks used in these two groups of studies, e.g., a shorter presentation time in our experiments. However, the most contributing factor, in our view, is that the ERPs in previous studies (Scholte et al., 2009; Groen et al., 2012a,b, 2013) were usually based on 2–4 averages of trials, whereas we used single trial ERPs for each image. Nevertheless, despite all these differences, our amplitude analysis results, showed that the significant correlation between ERP amplitude and Weibull contrast statistics also exists when images are from a more diverse set of images and are presented for a relatively shorter time.

In this study, we used several image statistics to quantify their effects on ERPs modulation of rapidly presented natural images. We found that Weibull contrast statistics, that is more biologically plausible, explained the variance of ERPs frequency and amplitude better compared to other statistics such as Fourier statistics of images. These findings support previous modeling studies (Ghebreab et al., 2009) that found a better performance for Weibull statistics in comparison to Fourier statistics (Ghebreab et al., 2009; Scholte et al., 2009).

Our analysis shed light on the correlation between modulation of frequency power in ERPs with low-level image statistics in humans. This correlation was significant, but it was not very strong and had a rapid time-course (it became significant at ~110 ms after stimulus onset, and peaked at 138 ms). So, these results may need further investigation in order to reveal the other contributing factors affecting the modulation of the frequency content of neural responses with image statistics. Nonetheless, our findings show that not only the amplitude of signals is modulated by these statistics, but also the frequency of signals is affected in response to Weibull contrast statistics. This emphasizes the significance of Weibull statistics in studying scene perception. Also, our results provide evidence for using the frequency content of signals, as a diagnostic feature, in future modeling studies. The phase of ERPs is another important property in which its relationship with image statistics can be investigated in future studies.

## Author contributions

Conceived and designed the experiments: MsG and MhG. Performed the experiments: MhG and MsG. Analyzed the data: MhG, MsG and AY. Contributed materials/analysis tools: MsG, MhG and AY. Wrote the paper: MsG and MhG.

### Conflict of interest statement

The authors declare that the research was conducted in the absence of any commercial or financial relationships that could be construed as a potential conflict of interest.

## Supplementary material

Figure S1 **Topographic representations for average correlation (*r*^2^), across electrodes and subjects, between beta parameter and ERPs amplitude for different time points**. Each topographic map shows the correlation between beta parameter and ERPs amplitude across time points and all channels. *r*^2^ were calculated for every channel and subject and then averaged out to generate topographic maps. The thick, horizontal, black line represents the stimulus presentation time. Note that the timing is not evenly spaced between 100 and 200 ms in order to highlight the maximum correlation time point.

Figure S2 **Regression analysis on ERPs**. Explained variance of ERP amplitude at channel Oz over time for *Full model* **(A)** and *Weibull model* **(B)**. Each individual subject is shown using thin lines and the mean across subjects is depicted using dark blue thick line. The significant correlation is illustrated using blue horizontal dots at the top of each plot for different times (FDR-corrected). The vertical, transparent band in each plot shows the timing for stimulus presentation. The shaded blue area is standard deviation (STD). Explained variance of ERP frequency at channel Oz for *Full model* **(C)** and *Weibull model* **(D)**.

Figure S3 **Topographic representations for average correlation (*r*^2^), across electrodes and subjects, between beta parameter and the power in different frequencies of ERPs**. Each topographic map shows the correlation between beta parameter and power in a specific frequency of ERP signals. The correlation values (*r*^*2*^) were calculated for every channel and subjects and then averaged out to generate topographic maps.

Video S1 **The modulation of EEG signals in response to natural images across different electrodes and time points**. The topographic representations show ERP modulations across different electrodes starting from 100 ms before stimulus onset to 600 ms after stimulus presentation. The color-coded topographic maps demonstrate the amplitude of signals specified with a color bar at the right end (the color bar shows positive and negative modulations in arbitrary units). The stimulus was presented at 0 ms and continued to ~70 ms.

Video S2 **Topographic representations for average correlation (*r*^2^) across electrodes and subjects for different time points**. The topographic representations show the correlation between beta parameter and ERPs amplitude for different time points starting from 100 ms before stimulus onset to 600 ms after stimulus presentation. The correlation values (*r*^2^) were calculated separately for every electrode and subject and then averaged out to generate topographic maps.
