# Supplementary figures and images for: Low-Level Contrast Statistics of Natural Images Can Modulate the Frequency of Event-Related Potentials (ERP) in Humans
Source: Front Hum Neurosci. 2016 Dec 9;10:630. doi: 10.3389/fnhum.2016.00630 (PMC5145888; doi:10.3389/fnhum.2016.00630)

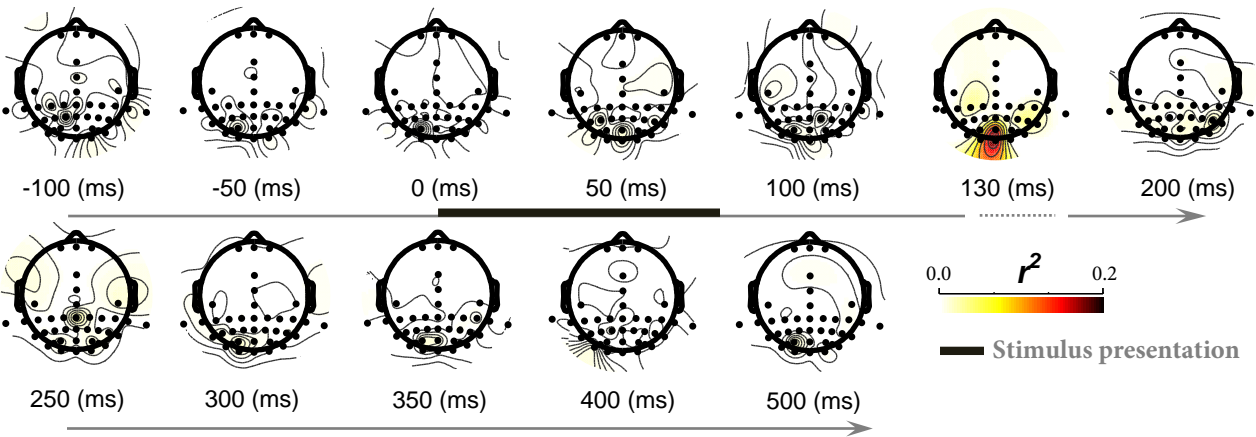

Supplement: Figure S1 — Topographic representations for average correlation (r2), across electrodes and subjects, between beta parameter and ERPs amplitude for different time points. Each topographic map shows the correlation between beta parameter and ERPs amplitude across time points and all channels. r2 were calculated for every channel and subject and then averaged out to generate topographic maps. The thick, horizontal, black line represents the stimulus presentation time. Note that the timing is not evenly spaced between 100 and 200 ms in order to highlight the maximum correlation time point. [file Image1.PDF]

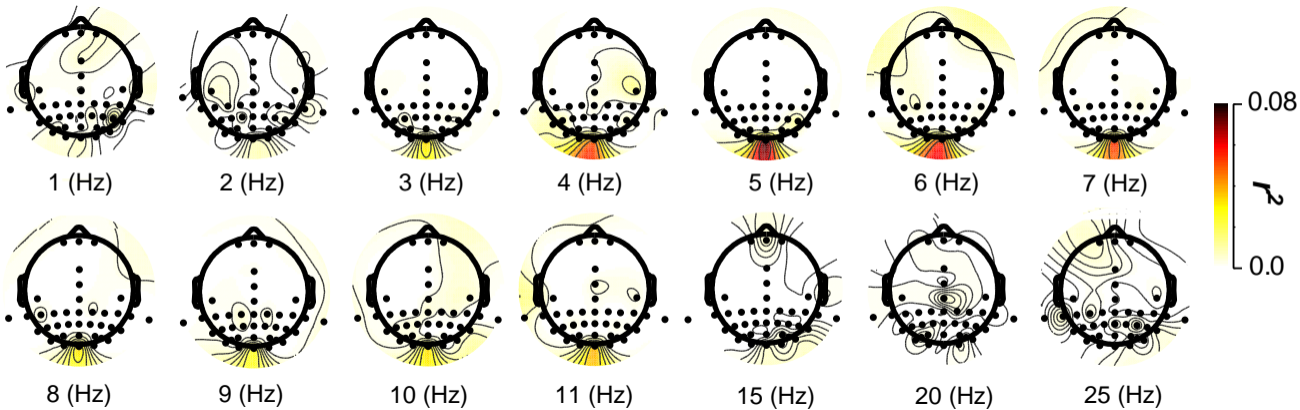

Supplement: Figure S3 — Topographic representations for average correlation (r2), across electrodes and subjects, between beta parameter and the power in different frequencies of ERPs. Each topographic map shows the correlation between beta parameter and power in a specific frequency of ERP signals. The correlation values (r2) were calculated for every channel and subjects and then averaged out to generate topographic maps. [file Image3.PDF]
