# Supplementary material for: Low-Level Contrast Statistics of Natural Images Can Modulate the Frequency of Event-Related Potentials (ERP) in Humans
Source: Front Hum Neurosci. 2016 Dec 9;10:630. doi: 10.3389/fnhum.2016.00630 (PMC5145888; doi:10.3389/fnhum.2016.00630)
Supplement: Figure S2 — Regression analysis on ERPs. Explained variance of ERP amplitude at channel Oz over time for Full model (A) and Weibull model (B). Each individual subject is shown using thin lines and the mean across subjects is depicted using dark blue thick line. The significant correlation is illustrated using blue horizontal dots at the top of each plot for different times (FDR-corrected). The vertical, transparent band in each plot shows the timing for stimulus presentation. The shaded blue area is standard deviation (STD). Explained variance of ERP frequency at channel Oz for Full model (C) and Weibull model (D). [file Image2.PDF]

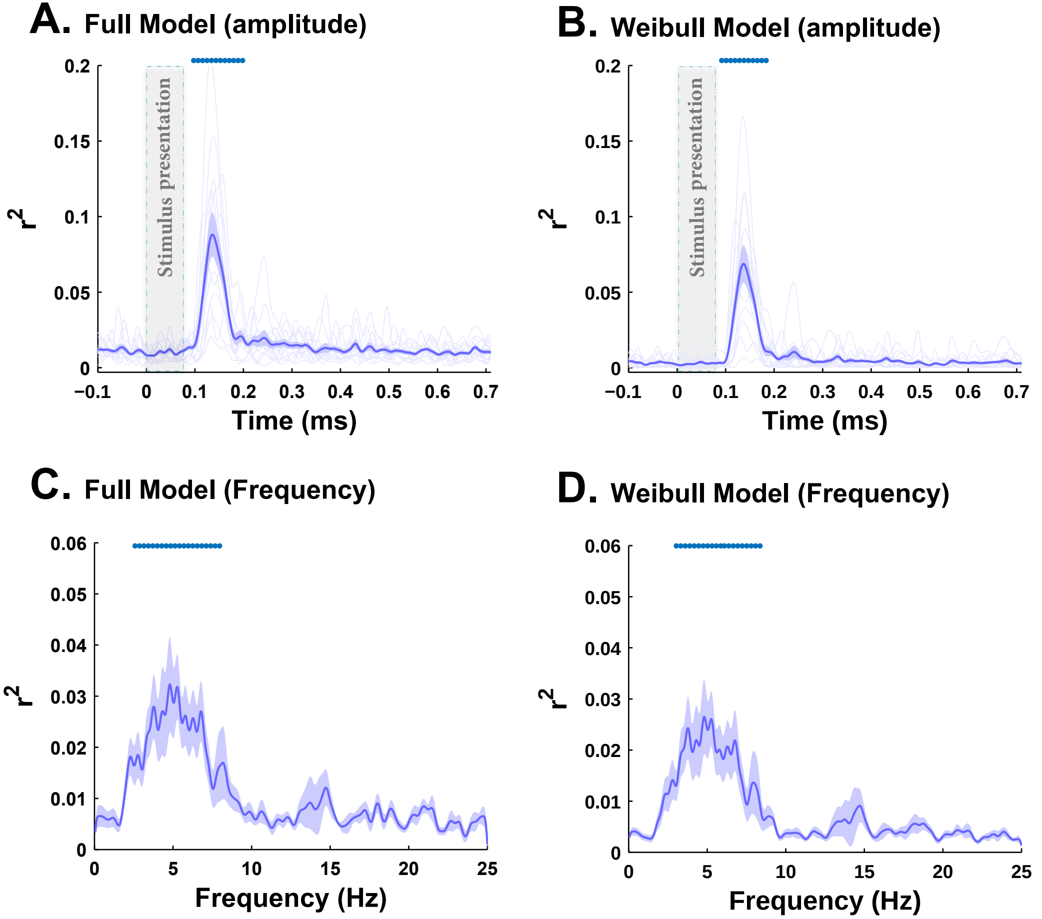

A. Full Model (amplitude)
B. Weibull Model (amplitude)
Stimulus presentation
Stimulus presentation
r2
0.2
0.15
0.1
0.05
0
−0.1
0
0.1
0.2
0.3
0.4
0.5
0.6
0.7
Time (ms)
Time (ms)
C. Full Model (Frequency)
D. Weibull Model (Frequency)
0.06
0.05
0.04
0.03
0.02
0.01
0
5
10
15
20
25
Frequency (Hz)
Frequency (Hz)
